# Fast and Adaptive Multi-Agent Planning under Collaborative Temporal Logic Tasks via Poset Products

**DOI:** 10.34133/research.0337

**Published:** 2024-03-20

**Authors:** Zesen Liu, Meng Guo, Weimin Bao, Zhongkui Li

**Affiliations:** ^1^ Department of Mechanics and Engineering Science, College of Engineering, Peking University, Beijing 100871, China.; ^2^ Science and Technology Commission of China Aerospace Science and Technology Corporation, Beijing 100048, China.

## Abstract

Efficient coordination and planning is essential for large-scale multi-agent systems that collaborate in a shared dynamic environment. Heuristic search methods or learning-based approaches often lack the guarantee on correctness and performance. Moreover, when the collaborative tasks contain both spatial and temporal requirements, e.g., as linear temporal logic (LTL) formulas, formal methods provide a verifiable framework for task planning. However, since the planning complexity grows exponentially with the number of agents and the length of the task formula, existing studies are mostly limited to small artificial cases. To address this issue, a new planning paradigm is proposed in this work for system-wide temporal task formulas that are released online and continually. It avoids two common bottlenecks in the traditional methods, i.e., (a) the direct translation of the complete task formula to the associated Büchi automaton and (b) the synchronized product between the Büchi automaton and the transition models of all agents. Instead, an adaptive planning algorithm is proposed, which computes the product of relaxed partially ordered sets (R-posets) on-the-fly and assigns these subtasks to the agents subject to the ordering constraints. It is shown that the first valid plan can be derived with a polynomial time and memory complexity with respect to the system size and the formula length. Our method can take into account task formulas with a length of more than 400 and a fleet with more than 400 agents, while most existing methods fail at the formula length of 25 within a reasonable duration. The proposed method is validated on large fleets of service robots in both simulation and hardware experiments.

## Introduction

Recent advances in computation, perception, and communication allow the deployment of autonomous robots in large, remote, and hazardous environments, e.g., to assist service staff in hospitals [1], to maintain offshore drilling platforms [2], and to monitor and assist construction sites [3]. Furthermore, fleets of heterogeneous robots, such as unmanned ground vehicles and unmanned aerial vehicles, are deployed to accomplish tasks that are otherwise too inefficient or even infeasible for a single robot [4]. Not only the overall efficiency of the team can be largely improved by allowing the robots to move and act concurrently [5] but also the capabilities of the team can be greatly extended by enabling multiple robots to directly collaborate on a task [6]. Recent works have demonstrated such potentials preliminary for simple tasks such as collaborative exploration [7], formation control [8], object transportation [9], and pursuer–evader games [10]. The task planning problem for multi-robot systems is in general NP-hard [11], due to the inherent combinatorial nature of robot-task assignment and various constraints such as capabilities and deadlines. The standard approach is to formulate a mixed integer linear programs (MILPs) over the integer variables and constraints [12]. While being sound and optimal, these methods are applicable to only small-scale systems. Thus, extensive work can be found on designing meta-heuristic algorithms for finding sufficiently good solutions in a reasonable time, e.g., genetic algorithms [13], colony optimization [14,15], particle swarm optimization [16,17], learning-based algorithms [18–20], or large language models [21,22]. However, these methods often lack a formal guarantee on the correctness and quality of the planning results.

Moreover, to specify more complex tasks, many recent works propose to use formal languages such as linear temporal logic (LTL) [23], computation tree logic (CTL) [24], and signal temporal logic (STL) [25] as an intuitive yet powerful way to describe both spatial and temporal requirements on global [26] or local [27] behaviors. Notably, the works in [28–30] formulate MILP by a central planning unit given different system models and task constraints; the works in [31–35] instead propose various search algorithms over the state or solution space of the whole system. However, the aforementioned planning methods are often executed offline for a set of predefined static tasks. A particularly challenging scenario is when the system operates indefinitely, i.e., new tasks are released or canceled dynamically and continually by external demand [36], or certain target features related to the tasks can change location during run time [10]. This would require the fleet to adaptively change their task plans online to modify existing assignments and incorporate new tasks. Thus, the aforementioned methods become inadequate as the sequence of tasks is infinite and their specifications are unknown beforehand. Recursive application of the centralized methods in a naive way leads to not only intractable computation complexity but also inconsistent or even oscillatory assignments. Thus, an efficient and adaptive planning scheme is essential for multi-robot systems that collaborate in a dynamic environment [37–39] or an unknown environment [40].

### Related work

The standard framework for planning under temporal tasks is based on the model-checking algorithm [23]: First, the task formulas are converted to a deterministic Robin automaton (DRA) or nondeterministic Büchi automaton (NBA) via off-the-shelf tools such as SPIN [41] and LTL2BA [42]. Second, a product automaton is created between the automaton of formula and the models of all agents, such as weighted finite transition systems (wFTSs) [23], Markov decision processes (MDPs) [43], or petri nets [44]. Last, certain graph search or optimization procedures are applied to find a valid and accepting plan within the product automaton, such as nested-Dijkstra search [32], integer programs [45], auction [46], or sampling-based [33,47].

Thus, the fundamental step of all aforementioned methods is to translate the task formula into the associated automaton. This translation may lead a double-exponential size w.r.t. the formula length as shown in [42]. The only exceptions are GR(1) formulas [48], of which the associated automaton can be derived in polynomial time but only for limited cases. In fact, for general LTL formulas with length more than 25, it takes more than 2 h and 13 GB memory to compute the associated NBA via LTL2BA. Although recent methods have greatly reduced the planning complexity in other aspects, the length of considered task specifications remains limited due to this translation process. For instance, the sampling-based method [33,47] avoids creating the complete product automaton via rapidly exploring random tree (RRT) sampling, of which the largest simulated case has 400 agents and the task formula has a maximum length of 14. The planning method [32] decomposes the resulting task automaton into independent subtasks for parallel execution. The simulated case scales up to 100 robots and a task formula of maximum length 18. Moreover, other existing works such as [49–52] mostly consider task formulas of length around 6 to 10. This limitation hinders its application to more complex robotic missions.

This drawback becomes even more apparent for dynamic scenes, where contingent tasks specified as LTL formulas are triggered by external observations and released to the whole team online. In such cases, most existing approaches compute the automaton associated with the new task, of which the synchronized product with the current automaton is derived, see, e.g., [51,52]. Thus, the size of the resulting automaton equals to the product of all automata associated with the contingent tasks, which is clearly a combinatorial blow-up. Consequently, the amount of contingent tasks that can be handled by the aforementioned approaches is limited to handpicked examples.

### Our contribution

To overcome this curse of dimensionality in the size of tasks and agents, we propose a new paradigm that is fundamentally different from the model checking-based methods. First, for a syntactically co-safe LTL (sc-LTL) formula that is a conjunction of numerous subformulas, we calculate the R-posets of each subformula as a set of subtasks and their partial temporal constraints. Then, an efficient algorithm is proposed to compute the product of R-posets associated with each subformula. The resulting product of R-posets is complete in the sense that it retains all subtasks from each R-poset along with their partial orderings and resolves potential conflicts. Given this product, a task assignment algorithm called the time-bound contract net (TBCN) is proposed to assign collaborative subtasks to the agents, subject to the partial ordering constraints. Last but not least, the same algorithm is applied online to dynamic scenes where contingent tasks are triggered and released by online observations. It is shown formally that the proposed method has a polynomial time and memory complexity to derive the first valid plan w.r.t. the system size and formula length. Extensive large-scale simulations and experiments are conducted for a hospital environment where service robots react to online requests such as collaborative transportation, cleaning, and monitoring.

The main contribution of this work is threefold: (a) a systematic method is proposed to tackle task formulas with length more than 400, which overcomes the limitation of existing translation tools that can only process formulas of length less than 25 in reasonable time; (b) an efficient algorithm is proposed to decompose and integrate contingent tasks that are released online, which not only avoids a complete re-computation of the task automaton but also ensures a polynomial complexity to derive the first valid plan; (c) the proposed task assignment algorithm is fully compatible with both static and dynamic scenarios with interactive objects.

### Problem statement

Consider a multi-agent system with heterogeneous capabilities, a series of sc-LTL task formulas that are released online, and a set of interactive objects in the dynamic environment. The objective is to generate a task plan for the system online such that these tasks are satisfied with high efficiency.

For instance, as shown in the “Numerical simulation” section, a fleet of heterogeneous service robots is deployed in the hospital environment. Different tasks such as transportation of goods or patients, cleaning, and maintenance are released online continuously. Many of such tasks contain numerous subtasks with ordering constraints that require direct collaboration of different robots. An efficient coordination algorithm is proposed such that these subtasks are assigned and fulfilled online in a timely manner.

## Results

In this section, we present the proposed solution briefly, where we first give a definition of task specification and introduce the core method of computing the R-poset product. Then, an efficient assignment algorithm is introduced under these posets with temporal and spatial constraints. The simulation and hardware experiment results are presented against several strong baselines for different sizes of fleets and task complexities. Technical details and derivations can be found in Materials and Methods.

### Task specification and R-poset product

We briefly introduce the syntax of LTL used for task specifications. The basic ingredients of LTL formulas are a set of atomic propositions *AP* in addition to several Boolean and temporal operators. Atomic propositions are Boolean variables that can be either true or false. The syntax of LTL is defined as *φ* ≜ ⊤  ∣ *p* ∣ *φ*_1_ ∨ *φ*_2_ ∣ *φ*_1_ ∧ *φ*_2_ ∣ ¬ *φ* ∣ ◯ *φ* ∣ *φ*_1_ *Uφ*_2_ ∣ ◻ *φ* ∣ ⋄ *φ*, where ⊤ ≜ True; *p* ∈ *AP* is the alphabet; ∨ (conjunction), ∧ (disjunction), and ¬ (negation) are the logical operators; ⋄ (eventually), ◯ (next), and *U* (until) are the temporal operators; and ◻ (always) and ⇒ (implication) are the derived operators. A special class of LTL formula called sc-LTL [23,53] only contains the temporal operators ◯, *U*, and ⋄. A complete description of the semantics and syntax of LTL can be found in [23].

Given a series of sc-LTL formulas that are released online, most existing methods would combine the formulas with ∧ (conjunction) and convert them into an NBA. It results in a graph structure that consists of states, transitions, guards, inital states, and final states. However, since its size is double exponential to the length of formula *φ* as proven in [42], it quickly becomes intractable with more task formulas. Instead, we compute the product of R-posets associated with these formulas, called the Poset-prod (denoted by ⊗). The R-poset *P* = (Ω,  ⪯ , ≠) is a high-level abstraction of NBA, proposed in our earlier work [54], which consists of a set of subtasks Ω and their partial relations as less equal ⪯ and conflict ≠. It has been proven therein that if the subtasks are executed under the partial relations, the resulting traces satisfy the NBA. Once a new task formula is released, it is transformed into a new R-poset *P*_2_ and its product with the current R-poset *P*_1_ is computed. More specifically, given *P*_1_, *P*_2_, the Poset-prod returns a new set of R-posets $P=P_{1}⊗P_{2}$ that satisfies both *P*_1_ and *P*_2_, which is computed by iterating the following two procedures: Task composition and Relation update. The first procedure is to create a group of subtasks as a combination between the subtasks in *P*_2_ and the subtasks of *P*_1_ that have not been executed. A depth-first-search (DFS) algorithm is proposed to gradually add the subtasks along with the corresponding mapping function. The second procedure is to calculate the partial relations between the subtasks such that the ordering constraints among the subtasks in the composed product are consistent without conflicts. Consequently, the overall poset is given by *P_final_* = (Ω*_f_*, ⪯*_f _*, ≠*_f_*), which is iteratively computed each time a new poset is added.

The proposed Poset-prod method outperforms the traditional method in both computational efficiency and performance. This improvement becomes even more pronounced when the number and length of subformulas increase. This is because the time of generating NBAs of *φ*_1_, *φ*_2_, ⋯ grows linearly, while the time of converting *φ*_1_ ∧ *φ*_2_ ∧ ⋯ into NBA grows exponentially. Moreover, for a new added formula, the algorithm updates the final R-poset based on the previous result, which ensures the performance for online cases. Finally, it is an anytime algorithm that the algorithm can calculate an R-poset within linear complexity for multiple subformulas at the expense of optimality. The concrete complexity analysis of Poset-pord is shown in Discussion, and the definition of R-poset and label is shown in Materials and Methods.

**Fig. 1.**
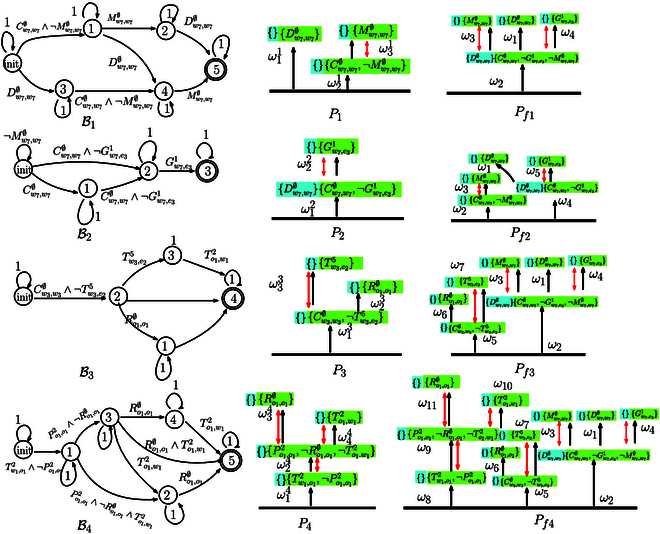
Example of Poset-prod associated with four formulas $\varphi_{1}=⋄D_{w7,w7}^{\emptyset }\wedge ⋄\left(C_{w7,w7}^{\emptyset }\wedge \neg M_{w7,w7}^{\emptyset }\wedge ⋄M_{w7,w7}^{\emptyset }\right)$, $\varphi_{2}=⋄\left(C_{w7,w7}^{\emptyset }\wedge \neg G_{w7,e3}^{1}\wedge ⋄G_{w7,e3}^{1}\right)\wedge \neg D_{w7,w7}^{\emptyset }UC_{w7,w7}^{\emptyset }$, $\varphi_{3}=⋄$$\left(C_{w3,w3}^{\emptyset }\wedge \neg T_{w3,e2}^{5}\wedge ⋄D_{w3,w3}^{\emptyset }\wedge ⋄T_{w3,e2}^{5}\right)$, and $\varphi_{4}=⋄\left(T_{w1,o1}^{2}\wedge \neg P_{o1,o1}^{2}\wedge ⋄\left(P_{o1,o1}^{2}\wedge \neg R_{o1,o1}^{\emptyset }\wedge ⋄T_{o1,w1}^{2}\wedge ⋄R_{o1,o1}^{\emptyset }\right)\right)$. Left: $B_{1},\cdots ,B_{4}$ are the NBAs of formula *φ*_1_, ⋯, *φ*_4_. Middle: *P*_1_, ⋯, *P*_4_ are the R-posets of $B_{1},\cdots ,B_{4}$. Right: $B_{1},\cdots ,B_{4}$*P*_1_ ⊗ *P*_2_ = {*P*_*f*1_, *P*_*f*2_}, $P_{f_{3}}\in P_{f_{1}}⊗P_{3}\;\text{and}\;P_{f_{4}}\in P_{f_{3}}⊗P_{4}$.

To give an example, consider four subformulas *φ*_1_, *φ*_2_, *φ*_3_, *φ*_4_ as shown in Fig. 1. Their NBAs $B_{1},\cdots ,B_{4}$ can be transformed into four R-posets *P*_1_, ⋯, *P*_4_. The first round of Poset-prod is between *P*_1_ and *P*_2_ as *P*_1_ ⊗ *P*_2_ = {*P*_*f*1_, *P*_*f*2_}. Then, the following rounds of Poset-prod are performed between the results of the previous round and the next R-poset as *P*_*f*1_ ⊗ *P*_3_, of which the first solution is denoted by *P*_*f*3_. At the expense of some optimality, the algorithm can go to next round before all results of *P*_*f*2_ ⊗ *P*_3_ are generated, as it is an anytime algorithm. Finally, we can derive *P*_*f*4_ by computing *P*_*f*3_ ⊗ *P*_4_ as the final R-poset, which satisfies *P*_1_, ⋯, *P*_4_. Note that the time to generate the NBA associated with *φ*_1_, $\wedge_{i=1}^{2}\varphi_{i}$, $\wedge_{i=1}^{3}\varphi_{i}$, and $\wedge_{i=1}^{4}\varphi_{i}$ grows significantly with a duration of 0.058 s, 0.076 s, 1.56 s, and 25.56 s via LTL2BA [42]. By contrast, the time to compute these products *P*_1_ ⊗ *P*_2_, *P*_1_ ⊗ *P*_2_ ⊗ *P*_3_, *P*_1_ ⊗ *P*_2_ ⊗ *P*_3_ ⊗ *P*_4_ grows slower with a duration of 0.199 s, 0.279 s, and 0.385 s. Thus, our method can deal with a much larger number of tasks online. Detailed comparisons between our method and traditional methods can be found in the “Scalability analysis and comparisons” section.

### Online subtask assignment under complexity constraints

**Fig. 2.**
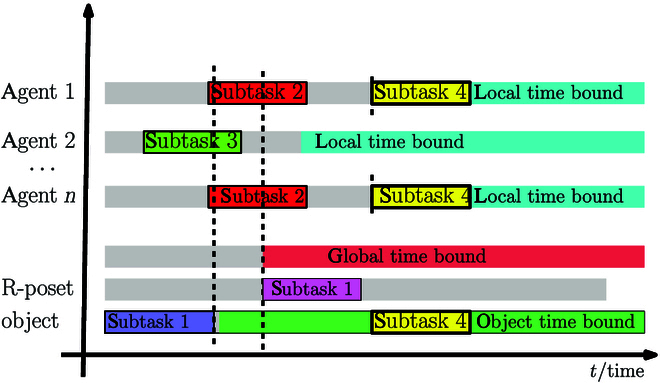
The bidding process of assigning subtask 4 under the global time bound (when the partial relations in R-poset are satisfied), the time bound for object (when the object is reachable), and local time bounds (when the agents get ready).

Once the set of R-posets that satisfies all subformulas is generated, a series of subtasks in the R-poset should be assigned to the agents under several complexity constraints, including temporal constraints from R-poset, objects constraints, and cooperative constraints. Similar to the classical contract net method [55], we propose an efficient and suboptimal assignment algorithm called time bound contract net (TBCN). The main difference is that all these constraints are represented uniformly by time bounds in our methods, and an example is shown in Fig. 2. The final assignment is a group of timed sequence of robot actions $J=\left[J_{1}\;,\;\cdots \;,\;J_{n}\right]$, where *J_n_* = [(*t_k_*, *ω_k_*, *a_k_*), ⋯] indicates that agent *n* will execute action *a_k_* at time *t_k_* to satisfy subtask *ω_k_* such that the newly released tasks are fulfilled.

TBCN consists of three steps: initialization, computation of feasible subtasks, and online bidding. As the partial constraints of final R-poset *P_final_* might be changed after computing the product, the subtasks that are conflicting with the updated partial orders are removed in initialization. Then, the last two steps are iterated: the set of subtasks whose partial orders are satisfied given the current assignment J in computation of feasible subtasks. Consequently, a linear program is formulated and solved in the online bidding, to choose one subtask from the feasible set. Thus, the resulting execution time and action plans are added to J.

### Numerical simulation

As shown in Fig. 4, the hospital environment consists of the wards, the operating rooms, the hall, the exits, and the hallways. The multi-agent system is employed with three junior doctors, six senior doctors, and eight nurses, and four types of patients are treated as interactive objects including family visitors, vomiting patients, senior patients, and junior patients. The detailed mappings between agents, action, objects, and their labels are shown in Table S1. Furthermore, various types of tasks are considered, such as “Go the rounds of the wards and provide medicine,” “Check and record the patient status,” and “Prepare and perform an operation on a patient.” Some tasks are released online under certain conditions, for instance, when a patient vomits at a region, the task “Take the patient into ward and check his status. Meanwhile, the doctors should not enter this region until it has been cleaned.” The sc-LTL formula associated with the complete task is given by $\begin{array}{c} \varphi =\varphi_{b1}\wedge \cdots \varphi_{b_{6}}\wedge \varphi_{\mathrm{VP}}\wedge \end{array}$$\begin{array}{c} \varphi_{\mathrm{JP}}\wedge \varphi_{\mathrm{SP}}^{1}\wedge \varphi_{\mathrm{SP}}^{2}\wedge \varphi_{\mathrm{FV}} \end{array}$, which has a total length of 62. Detailed description of formulas are shown in Table S2.

**Fig. 3.**
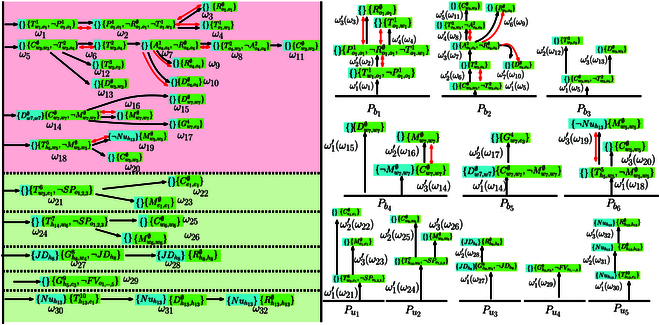
Illustration of computing the product posets. Left: Final R-poset computed from the initial subformulas and online subformulas (dashed lines). Right: $P_{b_{1}},\;\cdots \;,P_{b_{6}}$ are the R-posets associated with the initial subformulas, and $P_{u_{1}},\;\cdots \;,P_{u_{5}}$ are associated with the online subformulas.

As shown in Fig. 3, each subtask *ω_i_* consists of the constraints before execution (in blue) and during execution (in green). The directed black arrows denote the ordering constraints, while the bidirectional red arrows for the conflicting constraints. The mapping from the subtasks within the input R-posets to the subtasks within the final R-poset is shown in brackets as $\omega_{1}^{\prime }(\omega_{1}^{\prime }):\omega_{1}^{\prime }$ → *ω*_1_. Note that most of R-posets on the right are directly incorporated into the final R-posets on the left, such as $P_{b_{1}},\;\cdots \;,P_{b_{6}},P_{u_{1}},\;\cdots \;,P_{u_{5}}$. This is due to the fact that their subtasks are independent without ordering constraints, which allows for parallel execution. Additionally, some subtasks on the left represent the same subtasks on the right. For example, $\omega_{1}^{\prime }$ of $P_{b_{2}}$ and $\omega_{1}^{\prime }$ of $P_{b_{2}}$ representing both *ω*_5_, since their ordering constraints (in green) are identical. The same action can be performed to satisfy multiple subtasks on the left, which improves the overall efficiency. Furthermore, all subtasks on the right satisfy the partial relations between the corresponding subtasks on the left, while additional constraints are added if there are conflicts among the ordering constraints. For instance, action $D_{w_{7},w_{7}}^{\emptyset \emptyset }$ of *ω*_15_ should not be executed before the subtask *ω*_14_. Thus, an additional ordering constraint is added such that subtask *ω*_15_ should be executed after *ω*_14_. These properties guarantee that each subtask within the R-posets on the left can be executed, as their partial relations are satisfied. Consequently, the final R-poset satisfies all R-posets on the left. Note that the complete formula has a total length of 62, of which the NBA takes more than 1 h to compute. On the contrary, the first final R-poset is computed within 10.96 s.

**Fig. 4.**
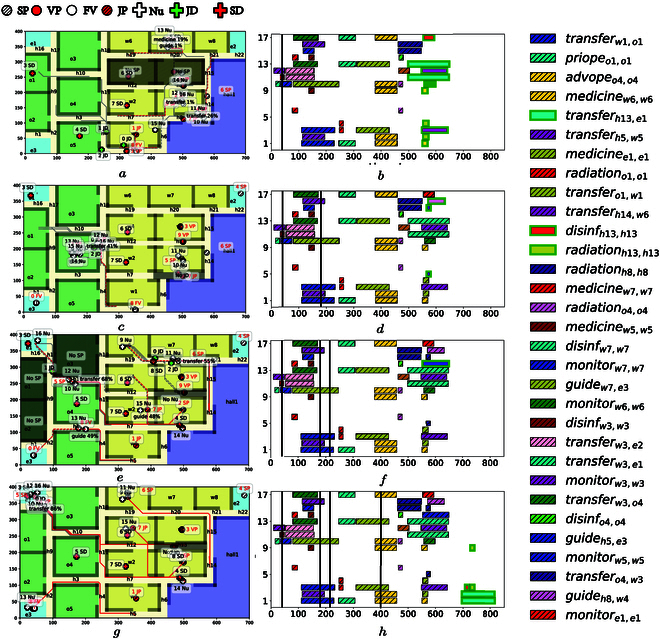
(A, C, E,and G): Snapshot of agent trajectories at 40, 180, 215, and 400 s when new tasks are added online. (B, D, F, and H): Gantt graph of task assignment at these time instants, as highlighted in green boxes.

The results of task assignment are shown in Fig. 4, which include the updates at 40, 180, 215, and 400 s with five additional objects added. Then, after modifying the current R-posets to accommodate these formulas, the method TBCN is executed to assign the new subtasks within the final R-poset. The trajectories of agents and objects are shown, with certain regions that are not allowed to enter. For instance, when a patient vomits at region h13 in Fig. 4C, the nurses cannot enter until other agents have cleaned this region. These constraints ensure that both their actions and their trajectories satisfy the R-poset. In addition, once a new object is added, a new formula is released and then the R-poset is updated. All subtasks satisfy the ordering constraints in the R-posets. For instances, as shown in Fig. 4D, although agents 6, 14 have arrived in region w7 before 100 s to collaborate on the subtask $\sigma_{16}=\left\{M_{w7,w7}^{\emptyset }\right\}$ (in blue), they have to wait until that agent 10 has fulfilled the subtask $\sigma_{14}=\left\{C_{w7,w7}^{\emptyset }\right\}$, due to the ordering constraints that (*ω*_14_, *ω*_16_) ∈  ⪯ , {*ω*_14_, *ω*_16_} ∈ ≠. The constraints introduced by objects are also satisfied, e.g., task *ω*_7_ (in green) cannot be executed at 380 s before subtask *ω*_6_ (in purple) has been finished, since the required object 3 has not been transferred to region *o*_4_. Last but not least, most tasks are executed in parallel mostly with a total completion time of 815 s, which is much shorter than 2013.5 s if the subtasks are executed sequentially.

### Scalability analysis and comparisons

**Fig. 5.**
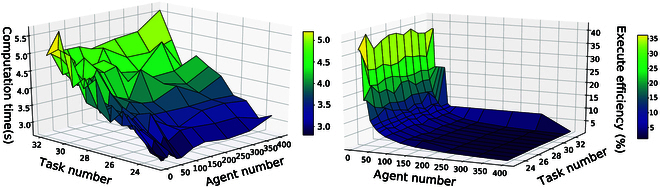
The computation time (left) and the execution efficiency *η* (right) with respect to different number of agents and subtasks.

First, we show how the computation time of the proposed task assignment method TBCN varies under different numbers of agents and subtasks. Since the number of subtasks cannot be directly determined, we run the TBCN with a large number of formulas, of which the length ranges from 20 to 80. The number of subtasks and the associated computation time are recorded. As shown in Fig. 5, the average computation time only increases slightly as the number of agents is increased from 12 to 400, while the computation time increases considerably if the number of subtasks is increased from 22 to 34. This is due to the fact that new subtasks would introduce additional temporal constraints in the assignment procedure. The execution efficiency *η* from Eq. 6 decreases as the number of agent increases, while *η* increases slightly as the number of subtasks grows. The highest *η* is about 39% where most subtasks are executed in parallel with only minimum waiting time for task synchronization.

**Fig. 6.**
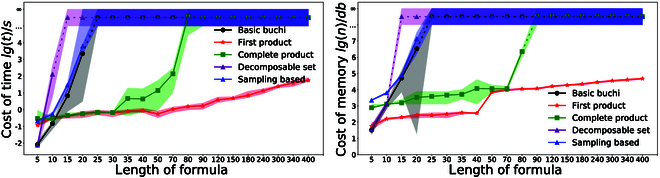
The comparison of the computation time (left) and memory (right) by different methods.

Second, to further validate the scalability of the proposed method against existing methods, we evaluate the time and memory cost to compute both the first R-poset and the complete R-posets by the proposed Poset-prod, given the task formulas of different lengths. The conversion from an LTL formula to NBA is via LTL2BA. The following three baselines are considered: (a) the direct translation from the complete formula to NBA [42], in which the complete formula is the conjunction of all subformulas; (b) the decomposition set-based algorithm [56], which decomposes the NBA into independent subtasks; (c) the sampling-based method [33,47], which generates a product automaton much smaller than the complete one by sampling the product states of NBA and weighted transition systems (WTS) via RRT. Each method is tested three times with five formulas of the same length ranging from 5 to 400. As shown in Fig. 6, both the time and memory cost increase drastically with the formula length for all methods except the proposed Poset-prod to compute the first R-poset. In particular, when the formula length exceeds 15, the decomposition algorithm runs out of memory or time. The sampling-based method cannot generate a solution when the formula length exceeds 20. Since all the baseline methods require the translation to NBA first, it becomes intractable as the formula length exceeds 25. In contrast, the proposed method of Poset-prod can generate all R-posets with a formula length of 70 and the first R-poset even when the length reaches 400, which is consistent with our analyses in Discussion.

### Hardware experiment

We further tested the proposed method on hardware within a simplified hospital environment. The multi-agent system consists of 10 differential-driven mobile robots, with 3 *JD* in green, 3 *SD* in yellow, and 4 *Nu* in blue. The required tasks include “prepare and execute an advanced operation for patient 1 at operating room *o4*.” Similar to the “Numerical simulation” section, there are event-triggered tasks released online such as “when a patient vomits, take him to the ward, do not enter this region until it is cleaned.” The exact task description and formulas are shown in Table S3.

**Fig. 7.**
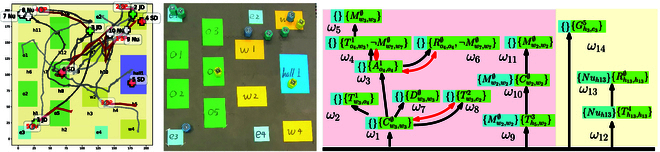
The trajectories of agents in experiment (left), experimental snapshot (middle), and the final R-poset (right).

**Fig. 8.**
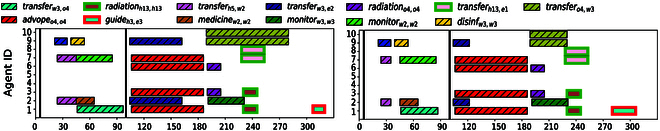
The Gantt graph of the planned execution (left) and the Gantt graph of the actual execution (right).

In summary, the system is required with six subformulas, and the final formula is *φ* = *φ*_*b*1_ ∧ ⋯ ∧$\varphi_{b_{4}}$ *φ_FV_* ∧ *φ_JP_*, with the total length 27; thus, it cannot be translated into an NBA directly. The agent trajectories within 185 s are shown in Fig. 7. As shown in the right of Fig. 7, the final R-poset consists of 14 subtasks, where the part in red is calculated offline and the part in yellow is generated online. Most subtasks are executed in parallel, meaning that the R-posets can be satisfied with a high efficiency. The complete R-poset product is derived in 3.1 s, and the task assignment method TBCN is activated three times, of which the average planing time is 2.7 s. As shown in the left of Fig. 8, the Gantt graph is updated twice at 5 and 100 s during execution, and subtasks that are released online are marked by green boxes. It is worth noting that the agent movement during real execution requires more time due to motion uncertainty, communication delay, drifting, and collision avoidance. Nonetheless, the proposed method can adapt to these fluctuations and still satisfy the specified tasks, as shown in the Gantt graph of the overall execution in Fig. 8. Experiment videos are provided in the Supplementary Materials.

## Discussion

In this work, we propose Poset-prod, an efficient online task planning algorithm for multi-agent systems where tasks are released dynamically and constantly online. It consists of a systematic method to convert the temporal tasks into their equivalent R-posets, of which their products are computed online. Given these R-posets, an anytime task assignment algorithm is proposed to adapt the local plans of robots online such that the overall safety and correctness are ensured. The overall framework is shown to be fast and efficient, thus particularly suitable for large-scale multi-agent systems collaborating in dynamic environments.

The most significant advantage of Poset-prod is that the complexity of obtaining the first solution only grows linearly with the length of formulas. Given a set of formulas {*φ_i_*, *i* ≤ *m*} and max*_i_* ∣ *φ_i_* ∣  = *n*, deriving the first solution has a polynomial complexity of *O*(*m*^2^*n*^3^). Despite that the overall complexity to compute the complete R-posets of {*φ_i_*} is *O*(*n*^5*m*^), it is still much smaller than the complexity of calculating the NBA of the conjunction *φ* via LTL2BA [42], which is *O*(*mn* · 2*^mn^*). Thus, our method can plan for task formulas with a length of about 400 within about 50 s, while most existing methods fail at the formula length of 25.

Future work involves two directions: (a) extending the sc-LTL task formulas to general LTL and other languages such as CTL [24] and STL [25]. It remains unclear how general LTL formulas with always operators can be incorporated in the R-poset, especially with the prefix–suffix structure, and (b) considering unknown and uncertain environments that are modeled as MDPs. In this case, a reactive high-level plan is essential to take into account all possible environment behaviors.

## Materials and Methods

In this section, we provide the knowledge of LTL in the “Preliminaries” section, the definition of alphabets and objective function in the “Problem formulation” section, and algorithm details in the “Approach” section.

### Preliminaries

As mentioned in the “Task specification and R-poset product” section of Results, the basic ingredients of LTL formulas are a set of atomic propositions *AP* in addition to several Boolean and temporal operators. For a given LTL formula *φ*, there exists an NBA as follows:

**Definition 1** An NBA $A≜\left(S,\;\Sigma ,\;\delta ,\left(S_{0},\;S_{F}\right)\right)$ is a four-tuple, where *S* are the states; *Σ* = *AP*; *δ* : *S* × *Σ* → 2*^S^* are transition relations; *S*_0_, *S_F_* ⊆ *S* are initial and accepting states.

An infinite word *w* over the alphabet 2*^AP^* is defined as an infinite sequence *W* = *σ*_1_*σ*_2_⋯, *σ_i_* ∈ 2*^AP^*. The language of *φ* is defined as the set of words that satisfy *φ*, namely, $L\left(\varphi \right)=\text{Words}\left(\varphi \right)=\left\{W\;,|,\;W⊨\varphi \right\}$ and ⊨ is the satisfaction relation. Additionally, the resulting run of *w* within A is an infinite sequence *ρ* = *s*_0_*s*_1_*s*_2_⋯ such that *s*_0_ ∈ *S*_0_, and *s_i_* ∈ *S*, *s*_*i*+1_ ∈ *δ*(*s_i_*,  *σ_i_*) hold for all index *i* ≥ 0. A run is called accepting if it holds that inf(*ρ*) ∩ *S_F_* ≠ ∅, where inf(*ρ*) is the set of states that appear in *ρ* infinitely often. A special class of LTL formula is called sc-LTL [23,53], which can be satisfied by a set of finite sequence of words. They only contain the temporal operators ◯, *U*, and ⋄ and are written in positive normal form where the negation operator ¬ is not allowed before the temporal operators. A relaxed partially ordered set (R-poset) over an NBA $B_{\varphi }$ is defined as follows:

**Definition 2 (R-poset)** [54] R-poset is a three-tuple: *P_φ_* = (*Ω_φ_*, ⪯*_φ_*, ≠*_φ_*)*:* $\begin{array}{c} \Omega_{\varphi }=\left\{\left(\ell ,\sigma_{\ell },\sigma_{\ell }^{s}\right),\forall_{\ell }=0,\ldots ,L\right\} \end{array}$ is the set of subtasks, where *ℓ* is the index of subtask *ω_ℓ_; σ_ℓ_* ⊆ *Σ* are the transition labels; $\sigma_{\ell }^{s}\subseteq \Sigma$ are the self-loop labels from Definition 1.

⪯*_φ_* ⊆ Ω*_φ_* × Ω*_φ_* is the “less equal” relation: If (*ω_h_*, *ω_ℓ_*) ∈ ⪯*_φ_* or equivalently *ω_h_*⪯*_φ_ω_ℓ_*, then *ω_ℓ_* can only be started after *ω_h_* is started. ≠*_φ_* ⊆ 2^Ω*_φ_*^ is the “opposed” relation: If {*ω_h_*, ⋯, *ω_ℓ_*} ∈ ≠*_φ_* or equivalently *ω_h_*≠*_φ_*⋯≠*_φ_ω_ℓ_*, subtasks in *ω_h_*, ⋯, *ω_ℓ_* cannot all be executed simultaneously.

A word is accepting if it satisfies all the constraints imposed by *P_φ_*. Additionally, the word of R-poset will satisfy the NBA as *Words*(*P_φ_*) ⊂ *Words*(*φ*).

**Definition 3 (Language of R-poset)** [54] Given a word *w* = *σ*′_1_*σ*′_2_⋯ satisfying R-poset *P_φ_* = (*Ω_φ_*, ⪯*_φ_*, ≠*_φ_*), denoted as *w* ⊨ *P_φ_*, it holds that (a) given $\omega_{i_{1}}=\left(i_{i},\sigma_{i_{1}},\sigma_{i_{1}}^{s}\right)\in \Omega_{\varphi }$, there exist *j*_1_ with $\sigma_{i_{1}}\subseteq \sigma_{j_{1}}^{\prime }$and $\sigma_{j_{1}}^{s}\cap \sigma_{m_{1}}^{\prime }=\emptyset ,\forall m_{1}<j_{1}$; (b) $\begin{array}{c} \forall \left(\omega_{i_{1}},\omega_{i_{2}}\right)\in \end{array}$ $\begin{array}{c} {⪯}_{\varphi },\exists j_{1}\leq j_{2},\sigma_{i_{2}}\subseteq \sigma \prime_{j_{2}},\forall m_{2}<j_{2},\sigma_{j_{2}}^{s}\subseteq \sigma \prime_{m_{2}}=\emptyset \end{array}$; (c) $\forall \left(\omega_{i_{1}},\ldots ,\omega_{i_{n}}\right)$ ∈≠φ,∃ℓ≤n,σiℓ⊆σ′j1. Language of R-poset *P_φ_* is the set of all word *w* that satisfies *P_φ_*, denoted by $L\left(P_{\varphi }\right)≜\left\{w,|,w⊨P_{\varphi }\right\}$*.*

Assuming that $P_{b_{i}}=\left\{P_{1}^{b_{i}},P_{2}^{b_{i}},\cdots \right\}$ is the set of all possible R-posets of NBA $B_{b_{i}}$, it is shown in Lemma 3 of our previous work [54] that (a) $L\left(P_{j}^{b_{i}}\right)\subseteq L\left(B_{b_{i}}\right),P_{j}^{b_{i}}\in P_{\varphi }^{b_{i}}$ and (b) ${⋃}_{P_{j}^{b_{i}}\in P_{\varphi }^{b_{i}}}‍L\left(P_{i}\right)=L\left(B_{b_{i}}\right)$. In other words, the R-posets contain the necessary information for subsequent steps.

### Problem formulation

#### Collaborative multi-agent systems

Consider a workspace $W\subset {\mathbb{R}}^{2}$ with *M* regions of interest denoted as W ≜ {*W*_1_, ⋯,*W_M_*} , where *W_m_* ∈ *W*. Furthermore, there is a group of agents denoted by N ≜ {1, ⋯, *N*} with different types ***L*** ≜ {1, ⋯, *L*}. More specifically, each agent n∈N belongs to only one type *l* = *M_type_*(*n*), where $M_{\text{type}}:N\to L$. Each type of agents *l* ∈ ***L*** is capable of providing a set of different actions denoted by $A_{l}$. The set of all actions is denoted as $A_{a}=\cup_{l\in L}‍A_{l}=\left\{a_{1},\cdots \;,a_{n_{C}}\right\}$. Without loss of generality, the agents can navigate between each region via the same transition graph, i.e., $G=\left(W,\to_{G},d_{G}\right)$, where →⊆W×W represents the allowed transitions and $d_{G}:\to_{G}\to {\mathbb{R}}_{+}$ maps each transition to its duration.

Moreover, there is a set of interactive objects O  ≜ {*o*_1_, ⋯,*o_U_*}with several types T  ≜ {*T*_1_, ⋯,*T_H_*} scattered across the workspace *W*. These objects are interactive and can be transported by the agents from one region to another. An interactive object $o_{u}\in O$ is described by a three-tuple:

$$o_{u}≜\left(T_{h_{u}},t_{u},W_{u}^{P}\right),$$ (1)

where $T_{h_{u}}\in T$ is the type of object; *t_u_* ∈ *R*^+^ is the time when *o_u_* appears in workspace *W*; $W_{u}^{p}:R^{+}\to W$ is a function that $W_{u}^{p}\left(t\right)$ returns the region of *o_u_* at time *t* ≥ *t_u_*; and $W_{u}^{p}\left(t_{u}\right)\subseteq W$ is the initial region. Additionally, new objects appear in *W* over time and are then added to the set O. With a slightly abusive notation, we denote the set of initial objects $O_{\text{in}}$ that already exist in *W* and the set of online objects $O_{\mathrm{on}}$ that are added during execution, i.e., $O=O_{\text{in}}\cup O_{\mathrm{on}}$.

To interact with the objects, the agents can provide a series of collaborative behaviors $C≜\left\{C_{1},\cdots ,C_{K}\right\}$. A collaborative behavior $C_{k}\in C$ is a tuple defined as follows:

$$C_{k}≜\left(o_{u_{k}},\left\{(a_{i},n_{i}),i\in L_{k}\right\}\right)$$ (2)

where $o_{u_{k}}\in O\cup \left\{\emptyset \right\}$ is the interactive object if any; $a_{i}\in A_{a}$ is the set of cooperative actions required; 0 < *n_i_* ≤ *N* is the number of agents to provide the action *a_i_*; and ***L****_k_* is the set of action indices associated with the behavior *C_k_*. Also, *d_k_* denotes the execution time of *C_k_*.

A behavior can only be executed if the required object is at the desired region. Since objects can only be transported by the agents, it is essential for the planning process to find the correct order of these transportation behaviors. Related works [50,57] build a transition system to model the interaction between objects and agents, the size of which grows exponentially with the number of agents and objects.

#### Task specifications

Consider the following two types of atomic propositions: (a) $p_{m}^{l}$ is true when any agent of type *l* ∈ ***L*** is in region $W_{m}\in W$; $p_{m}^{r}$ is true when any object of type r∈T is in region $W_{m}\in W$. Let $\begin{array}{c} p≜\left\{p_{m}^{l},\;\forall W_{m}\in W,l\in L\right\}‍⋃‍\left\{p_{m}^{r},\;\forall W_{m}\in W,r\in R\right\} \end{array}$. (b) ${\left(C_{k}\right)}_{k_{1},k_{2}}^{u_{k}}$ is true when the collaborative behavior *C_k_* in Eq. 2 is executed with object *o_u_k__*, starting from region *W*_*k*_1__ and ending in region *W*_*k*_2__. Let $\begin{array}{c} c≜ \end{array}\{{\left(C_{k}\right)}_{k_{1},k_{2}}^{u_{k}},\forall C_{k}\in C,$ $o_{u_{k}}\in O,\forall W_{k_{1}},W_{k_{2}}\in W\}$. Given these propositions, the team-wise task specification is specified as an sc-LTL formula over {**p**, **c**}:

$$\varphi =\left(\underset{i\in I}{\wedge }\varphi_{b_{i}}\right)\wedge \left(\underset{o_{u}\in O_{\mathrm{on}}}{\wedge }\varphi_{e_{u}}\right),$$ (3)

where $\begin{array}{c} \left\{\varphi_{b_{i}},\;i=1,\ldots ,\;I\right\},\;\left\{\varphi_{e_{u}},\;o_{u}\;\in O_{on}\right\} \end{array}$ are two sets of sc-LTL formulas over {**p**, **c**}. *φ_b_i__* is specified in advance, while *φ_e_u__* is generated online when a new object *o_u_* is added to $O_{\mathrm{on}}$.

To satisfy the LTL formula *φ*, the complete action sequence of all agents is defined as

$$J=\left(J_{1},\;J_{2},\;\cdots \;,\;J_{N}\right),$$ (4)

where $J_{n}\in J$ is the sequence of $\left(t_{k},\;C_{k},\;a_{k}\right)$, which means that agent *n* executes behavior $C_{k}$ by providing the collaborative service $a_{k}\in A_{a}$ at time *t_k_*. In turn, the sequences of actions for an interactive object is defined as:

$$\begin{array}{l} J^{o}=\left(J_{1}^{o}\;,\;J_{2}^{o}\;,\;\cdots \;,\;J_{U}^{o}\right), \end{array}$$ (5)

where $J_{u}^{o}=\left(t_{k},\;C_{k}\right)\cdots$ is the sequence of tasks associated with object $o_{u}\in O$. The task pair $\left(t_{k},\;C_{k}\right)$ is added to $J_{u}^{o}$ if object *o_u_* joins behavior $C_{k}$ at time *t_k_*. Assuming that the duration of formula *φ_b_i__*, *φ_e_i__* from being published to being satisfied is given by *D_i_*, the average efficiency is defined as

$$\begin{array}{l} \eta ≜\frac{\sum_{C_{k}\in J}‍|L_{k}|d_{k}}{D_{i}}, \end{array}$$ (6)

which is the percentage of time when actions are performed.

**Problem 1** Given the sc-LTL formula *φ* defined in Eq. 3, synthesize and update the motion and action sequence of agents J and objects $J_{o}$ for each agent n∈N to satisfy *φ* and maximize execution efficient *η.*

Although maximizing the task efficiency of a multi-agent system is a classical problem, the combination of interactive objects, long formulas, and contingent tasks imposes new challenges in terms of exponential complexity [42,50] and online adaptation [51].

### Approach

**Fig. 9.**
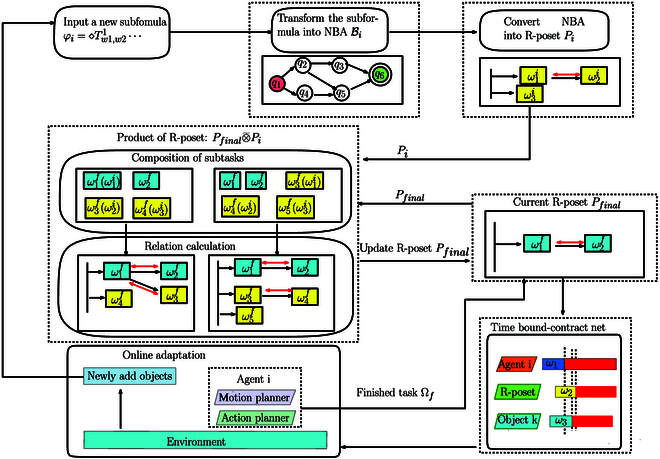
Framework of the proposed method. For a new input formula *φ_i_*, we first change it into an R-poset *P_i_* with the method in the “Preliminaries” section. Then, Poset-prod between *P_i_* and *P_final_* is calculated as in the “Product of R-posets” section. Third, after the R-poset *P_final_* is updated, the TBCN method in the “Task assignment” section determines and adjusts the task sequence of each agent and object. New tasks are released when the agents execute their local plans and detect new objects online.

As shown in Fig. 9, when a new R-poset is generated, the proposed solution realizes the requirement through two main components: (a) product of R-posets, where the product of existing R-posets is computed incrementally, and (b) task assignment, where subtasks are assigned to the agents given the temporal and spatial constraints specified in the R-poset.

#### Product of R-posets

As the first two steps shown in Fig. 9, when a new formula *φ_i_* ∈ *Φ_b_*, *Φ_u_* is added, it will be transformed into NBA first. Then, an R-poset *P_i_* is generated by the method proposed in our previous work [54]. The other R-poset *P_final_* involved in calculation is the previous result of Poset-prod, which will be updated after this round calculation. With *P_final_* as *P*_1_ = (Ω_1_, ⪯_1_, ≠_1_), *P_i_* as *P*_2_ = (Ω_2_, ⪯_2_, ≠_2_), we define product of R-posets as follows:

**Definition 4 (Product of R-posets)** Given a finite word *w*_0_, the product of two R-posets *P*_1_, *P*_2_ is defined as a set of R-posets $P^{r}=\{P_{1}^{\prime },P_{2}^{\prime },\cdots \}$, denoted as $P^{r}=P_{1}⊗P_{2}$ where $P_{i}^{\prime }$ satisfies two conditions: (a) if $w_{0}w\in L\left(P_{1}\right),w\in L\left(P_{2}\right)$, then $w_{0}w\in {⋃}_{P\prime_{i}\in P^{r}}‍L\left(P\prime \right)$; (b) if $w_{0}w\in L\left(P\prime_{i}\right)$*,* $P\prime_{i}\in P^{r}$, then $w_{0}w\in L\left(P_{1}\right),w\in L\left(P_{2}\right)$*.*

The word *w*_0_ is already executed, containing the finished subtasks $\Omega_{\text{finish}}^{1}$ of *P*_1_. Thus, *w*_0_ can not influence *P*_2_ whose subtasks will be executed in the future. Specially, if the new formula is offline as *φ_i_* ∈ *Φ_b_* and the agents have not started to execute subtasks, *w*_0_ will be set as empty. As shown in Fig. 9, Poset-prod consists of the following two steps.

(a) Task composition. In this step, we generate all possible combinations of subtasks that can satisfy both Ω_1_ and Ω_2_. Namely, a set of subtasks Ω′  = {$\omega_{1}^{\prime }$, ⋯,$\omega_{n}^{\prime }$} satisfies Ω_1_ if for each $\omega_{i}^{1}=(i,\sigma_{i}^{1},\sigma_{i}^{s1})\in \Omega_{1}$, there is a subtask $\omega_{j}^{\prime }=\left(j,\sigma_{j}^{\prime },\sigma_{j}^{s}\prime \right)\in \Omega \prime$ satisfying $\sigma_{i}^{1}\subseteq \sigma \prime_{j}$ and $\sigma_{i}^{s1}\subseteq \sigma_{j}^{s}\prime$, or $\omega \prime_{j}⊨\omega_{i}^{1}$ for brevity. Thus, we set Ω′ = Ω_1_ first and Ω′ clearly satisfies Ω_1_ with $\forall \omega_{i}^{1}\in \Omega_{1},\omega \prime_{i}⊨\omega_{i}^{1}$. Then, a mapping function *M*_Ω_ : Ω_2_ → Ω′ is defined to store the satisfying relationship from Ω_2_ to Ω′, and $D\left(M_{\Omega }\right)\in \Omega_{2}$ is the domain of *M*_Ω_ and $R\left(M_{\Omega }\right)$ is the range of *M*_Ω_. As all relations are unknown, *M*_Ω_, $D\left(M_{\Omega }\right)$, and $R\left(M_{\Omega }\right)$ are initially set to empty. Namely, if $\omega \prime_{j}⊨\omega_{i}^{2}$, we will store $M_{\Omega }\left(\omega_{i}^{2}\right)=\omega \prime_{j}$ and $M_{\Omega }^{-1}\left(\omega \prime_{j}\right)=\omega_{i}^{2}$, and add $\omega_{i}^{2}$ into $D\left(M_{\Omega }\right)$, and $\omega_{j}^{\prime }$ into *R*(*M*_Ω_), where $M_{\Omega }^{-1}$ is the inverse function of *M*_Ω_.

DFS [58] is used to add the subtasks of Ω_2_ to Ω′ in order, and these mapping relations will be recorded in *M*_Ω_. The search sequences of DFS can be initialized as *que* = [(Ω′ = Ω_1_, *M*_Ω_ = ∅)]. Then, during the circle, we will fetch the first node of que as (Ω′, $M_{\Omega }^{\prime }$). The next unmixed subtasks in Ω_2_ are $\omega_{i}^{2},i=|M\prime_{\Omega }|+1$. Any subtask $\omega_{j}^{1}\in \Omega_{1}/R\left(M\prime_{\Omega }\right)/\Omega_{\text{finish}}^{1}$ with $\omega_{j}^{1}⊨\omega_{i}^{2}$ or $\omega_{i}^{2}⊨\omega_{j}^{1}$ can create a new combination based on (Ω′, $M_{\Omega }^{\prime }$):

$$\begin{array}{c} \begin{array}{ll} \;\hat{\Omega }\prime & =\;\Omega \prime ,\;\hat{\omega }\prime_{j}=\left(j,\sigma_{j}^{1}\cup \sigma_{i}^{2},\;\sigma_{j}^{s1}\cup \sigma_{i}^{s2}\right),\\ \hat{M}\prime_{\Omega } & =\;M\prime_{\Omega },\;\hat{M}\prime_{\Omega }\left(\omega_{i}^{2}\right)=\;\omega \prime_{j}, \end{array} \end{array}$$ (7)

which means that the subtask $\omega_{j}^{\prime }$ in Ω′ can satisfy both $\omega_{j}^{1},\omega_{i}^{2}$. Moreover, for the subtask $\omega_{i}^{2}$, we can always create a set of subtasks $\hat{\Omega }\prime$ and the corresponding mapping function $\hat{M}\prime_{\Omega }$ by appending $\omega_{i}^{2}$ into $\hat{\Omega }\prime$ as $\hat{\omega }\prime_{j}$ such that $\hat{\omega }\prime_{j}⊨\omega_{i}^{2}$ holds, i.e.,

$$\begin{array}{l} \;\hat{\Omega }\prime =\Omega \prime ,j=|\Omega \prime |+1,\hat{\omega }\prime_{j}=\omega_{i}^{2};\\ \hat{M}\prime_{\Omega }=M\prime_{\Omega },\hat{M}\prime_{\Omega }\left(\omega_{i}^{2}\right)=\omega \prime_{j}, \end{array}$$ (8)

which means the subtask $\omega_{j}^{\prime }$ can be executed to satisfy $\omega_{i}^{2}$. This step ends if the time budget *t_b_* or the search sequence que exhausted. Once ∣*D*($M_{\Omega }^{\prime }$) ∣  =  ∣ Ω_2_∣, a Ω′ satisfying Ω_1_, Ω_2_ is already found. In this case, the next step is triggered. As shown in Fig. 9, one of found combination is $M_{\Omega }^{1}\left(1\right)=1,M_{\Omega }^{1}\left(2\right)=3,M_{\Omega }^{1}\left(3\right)=4$, $\omega_{1}^{\;f}$ is created by (7), and $\omega_{3}^{\;f},\omega_{4}^{\;f}$ is created by (8).

(b) Relation update. Given the set of subtasks Ω′ and the mapping function *M*_Ω_, we calculate the partial relations among them and build a product R-poset *P*. First, we construct the “less-equal” constraint ⪯ as follows:

$$\begin{array}{c} \begin{array}{l} ⪯={⪯}_{1}\cup \left\{\left(M_{\Omega }\left(\omega_{\ell_{1}}^{2}\right),M_{\Omega }\left(\omega_{\ell_{2}}^{2}\right)\right)|\left(\omega_{\ell_{1}}^{2},\omega_{\ell_{2}}^{2}\right)\in {⪯}_{2}\right\}, \end{array} \end{array}$$ (9)

which inherits both less equal relations ⪯_1_ in *P*_1_ and ⪯_2_ in *P*_2_. Then, we update Ω′ to consider the constraints imposed by the self-loop labels in other subtasks. Specifically, if a new relation (*ω_i_*, *ω_j_*) is added to ⪯ by $(M_{\Omega }^{-1}(\omega_{i})\;,\;M_{\Omega }^{-1}(\omega_{j}))\in {⪯}_{2}$ while (ωi1,ωj1)∈⪯1 holds, *ω_i_* is required to be executed before *ω_j_*, although $\left(\omega_{i}^{1},\;\omega_{j}^{1}\right)$ does not belong to ⪯_1_ of *P*_1_. In this case, *σ_i_* and $\sigma_{i}^{s}$ are updated to guarantee the satisfaction of the self-loop labels $\sigma_{j}^{s}$ before executing *σ_j_*. For each subtask *ω_i_*, the newly added suf-subtasks from ⪯_1_, ⪯_2_ are defined as $S_{p}^{1},S_{p}^{2}$, i.e.,

Sp1=ωj|ωi,ωj∈⪯,ωi1,ωj1∈⪯1,Sp2=MΩ−1ωj|ωi,ωj∈⪯,MΩ−1ωi2,MΩ−1ωj2∈⪯2, (10)

where* S^1^_p_, S^2^_p_* are the subtasks that should be executed after *ω_i_*. Thus, the action labels *σ_i_* and self-loop labels $\sigma_{i}^{s}$ in $\omega_{i}=(i,\;\sigma_{i},\;\sigma_{i}^{s})$ are updated accordingly as follows:

$$\begin{array}{l} \hat{\sigma }=\underset{\omega_{\ell }^{1}\in S_{p}^{1}}{⋃}‍\sigma_{\ell }^{s1}\cup \underset{\omega_{\ell }^{2}\in S_{p}^{2}}{⋃}‍\sigma_{\ell }^{s2},\sigma_{i}^{s}=\hat{\sigma }\cup \sigma_{i}^{s},\sigma_{i}=\hat{\sigma }\cup \sigma_{i}, \end{array}$$ (11)

in which $\sigma_{i}^{s}$ and *σ_i_* should be executed under the additional labels $\hat{\sigma }$; thus, the self-loop labels of $S_{p}^{1},S_{p}^{2}$ are satisfied.

Finally, we find the potential ordering by checking whether a subtask $\sigma_{i}^{s}$ is in conflict with another subtask while (ωi1, ωj1)∈⪯1. If so, an additional ordering will be added to ⪯ as:

⪯=⪯∪(ωi, ωj)|σj⊨σis (12)

Then, Ω will be updated following Eqs. 10 and 11. Regarding the set of subtasks Ω that have no conflicts in ⪯, its “not-equal” relations ≠ are generated by a simple combination as:

$$\begin{array}{l} \neq =\neq_{1}\cup \left\{\left\{M_{\Omega }\left(\omega_{\ell_{i}}\right)\right\}|\left\{\omega_{\ell_{i}}\right\}\in \neq_{2}\right\}. \end{array}$$ (13)

The resulting poset *P_i_* = (Ω,  ⪯ , ≠) is added to $P_{\text{final}}$.

As shown in Fig. 9, Relation update gets two R-posets and the partial relations of each R-poset are succeeded from *P_i_*, *P_final_*. Due to the anytime property, the two-step procedure can be repeated until all possible R-posets are found or just ended when the first R-poset is found.

#### Task assignment

To satisfy the final R-poset *P_final_* = (Ω*_f_*, ⪯*_f_*, ≠*_f_*), the subtasks Ω*_f_* should be executed under the partial orders of ⪯*_f_*, ≠*_f_*. Each subtask *ω_i_* ∈ Ω*_c_* represents a collaborative behavior *C_j_*. Thus, we can redefine the action sequence of each agent $J_{u}^{o}\in J_{o}$ as $J_{u}^{o}=[(t_{k},\;\omega_{k},a_{k}),\cdots \;]$ and the action sequence of each object $J_{u}^{o}\in J_{o}$ as $\begin{array}{c} J_{u}^{o}=[(t_{k},\;\omega_{k},\;a_{k}),\;\cdots \;] \end{array}$, in which we replace the cooperative behavior *C_k_* with *ω_k_* since *C_k_* ∈ *σ_k_*.

We propose a suboptimal algorithm called time bound contract net (TBCN) to generate and adapt the action sequence of agents and objects. Compared with the classical contract net method [55], the main differences are as follows: (a) the partial order ⪯*_f_*, ≠*_f_* of R-poset might be changed when new formula is added, (b) the assigned subtasks should satisfy the partial orders in ⪯*_f_*, ≠*_f_*, (c) the cooperative task should be fulfilled by multiple agents, and (d) interactive objects should be considered as an additional constraints. TBCN solves these differences with three steps: First, we design a cancellation mechanism in the initialization to adapt to the changes of R-poset mentioned in (a). Second, the partial orders in (b) are guaranteed by only assigning feasible subtasks but not all unassigned subtasks in each loop. Third, the constraints mentioned in (c) and (d) are considered as a time bound *t*_1_ ∈ ℝ^+^ in the bidding process.

(a) Initialization: Once the R-poset *P_final_* is updated by Poset-prod, we first collect the action sequence $J,J_{o}$ of previous solution and the set of finished subtasks Ω*_finish_* from executing word *w*_0_. Specially, all of them will be empty if it is the first round. Then, a set of essential conflict subtasks Ω*_ec_* is defined to collect the subtasks that might conflict the updated partial orders ⪯*_f_*, ≠*_f_*:

$$\begin{array}{c} \begin{array}{ll} \Omega_{\mathrm{ec}}= & \left\{\omega_{i}|\omega_{i}{⪯}_{f}\omega_{j},t_{i}\geq t_{j},\forall \omega_{i},\omega_{j}\in \Omega_{1}/\Omega_{\text{finish}}\right\}\cup \\ & \{\omega_{i}|\omega_{i}\neq_{f}\omega_{j},t_{j}\leq t_{i}\leq t_{j}+d_{j},\\ & \;\forall \omega_{i},\omega_{j}\in \Omega_{1}/\Omega_{\text{finish}}\}. \end{array} \end{array}$$ (14)

Then, we compute the set of subtasks Ω*_conf_* that should be removed from $J,J_{o}$:

$$\begin{array}{c} \begin{array}{ll} \Omega_{\text{conf}}= & \{\omega_{j}|\omega_{i}\;{⪯}_{f}\;\omega_{j},\forall \omega_{i}\in \Omega_{1}/\Omega_{\text{finish}},\\ & \forall \omega_{j}\in \Omega_{\mathrm{ec}}\}\cup \Omega_{\mathrm{ec}}, \end{array} \end{array}$$ (15)

in which Ω_*conf*_ are the subtasks in Ω*_ec_* and the subtasks whose pre-subtasks will be removed. With the action sequences $J,J_{o}$ removing all the subtasks in Ω*_conf_*, we can initialize the set of assigned subtasks $\begin{array}{c} \Omega_{\mathrm{as}}= \end{array}\{\omega_{i}|\forall (t_{i},\;\omega_{i},\;a_{i})\in J\}$ and the set of unassigned subtasks Ω*_u_* = Ω*_f_*/Ω*_as_*.

(b) Computation of feasible subtasks: After initialization, the algorithm starts a loop to assigned the subtasks in Ω*_u_*: getting a set of feasible subtasks Ω*_fe_*; calculating their time bounds; choosing the best subtask. For the ordering constraints ⪯*_c_*, if (*ω_j_*, *ω_i_*) ∈ ⪯*_c_*, assigning (*t_k_j__*, *ω_j_*, *a_k_j__*) to a task sequence *J_i_* = ⋯(*t_k_i__*, *ω_i_*, *a_k_i__*) will violate such constraints. Thus, Ω*_fe_* based on current Ω*_as_* is defined as:

$$\begin{array}{l} \Omega_{\mathrm{fe}}=\left\{\omega_{i}|\omega_{i}\in \Omega_{u},\forall \left(\omega_{j},\omega_{i}\right)\in {⪯}_{f},\omega_{j}\in \Omega_{\mathrm{as}}\right\}, \end{array}$$ (16)

in which the subtasks may lead to unfeasible action sequences being eliminated.

(c) Online bidding: Then, we will try assigning each subtask in Ω*_fe_* and only choose the one with the best result. Without loss of generality, we assume that subtask *ω_i_* ∈ Ω*_fe_* requires a label $\begin{array}{c} {\left(C_{k}\right)}_{k_{1},k_{2}}^{u_{k}} \end{array}$, which means that the agents need to execute the behavior *C_k_* from region *W*_*k*_1__ to region *W*_*k*_2__ using object *u_k_*. Any constraint mentioned in (b), (c), and (d) can be considered as a time bound *t*_1_ ∈ ℝ^+^, which means that such constraint can be satisfied after *t*_1_. Here, we use three kinds of time bounds: the global time bound $t_{i}^{\omega }$, the object time bound $t_{u_{k}}^{o}$, and the set of local time bounds ***T***_***s***_. The global time bound $\begin{array}{c} t_{i}^{\omega } \end{array}$ is the time instance that the ordering constraints ⪯*_f_* and conflict constraints ≠*_f_* will be satisfied if behavior ${\left(C_{k}\right)}_{k_{1},k_{2}}^{u_{k}}$ is executed after $t_{i}^{\omega }$:

$$\begin{array}{c} \begin{array}{ll} t_{i}^{\omega }\geq t_{j}, & \forall \left(j,i\right)\in {⪯}_{f},\omega_{j}\in \Omega_{\mathrm{as}},\\ t_{i}^{\omega }\geq t_{\ell }+d_{\ell }, & \forall \left\{\omega_{i},\omega_{\ell },\cdots \right\}\in \neq_{f},\omega_{\ell }\in \Omega_{\mathrm{as}}. \end{array} \end{array}$$ (17)

For the required object *u_k_*, assuming its participated last task is $J_{u_{k}}^{o}\left[-1\right]=\left(t_{\ell },\omega_{\ell }\right)$, the object time bound $t_{u_{k}}^{o}$ should satisfy that:

$$\begin{array}{l} W_{u}^{p}\left(t\right)=W_{k_{1}},t\geq t_{\ell }+d_{\ell },,\forall t\geq t_{u_{k}}^{o}, \end{array}$$ (18)

which means that the object *u_k_* will be at the starting region *W*_*k*_1__ of the current behavior $\begin{array}{c} {\left(C_{k}\right)}_{k_{1},k_{2}}^{u_{k}} \end{array}$ and ready for it after $t_{u_{k}}^{o}$. Additionally, we set $t_{u_{k}}^{o}=\infty$ =∞ if the object is not at *W*_*k*_1__ after the action sequence $J_{u_{k}}^{o}$, and we set $t_{u_{k}}^{o}=0$ if the behavior ${\left(C_{k}\right)}_{k_{1},k_{2}}^{u_{k}}$ does not require object as *u_k_* = ∅. The set of local time bound is defined as

$$\begin{array}{c} \begin{array}{l} T_{s}=\{\left(A_{n},t_{n}^{a}\right)|A_{n}=A_{M_{\text{type}}\left(n\right)}\cap A_{C_{k}},\\ t_{n}^{a}=t_{\ell }+d_{\ell }+d_{G}\left(k_{\ell },k_{1}\right),\forall n\;\in \;N\}, \end{array} \end{array}$$ (19)

where $A_{n}$ is the set of actions that agent *n* can provide for behavior *C_k_*, $t_{n}^{a}$ is the earliest time agent *n* can arrive region *W*_*k*_1__, *t_ℓ_* + *d_ℓ_* is the time when the last subtask *ω_ℓ_* ∈ *J_n_*−[1] has finished, $d_{G}\left(k_{\ell },k_{1}\right)$ is the cost of moving, and *W_k_ℓ__* is the goal region of *ω_ℓ_*. $\left(A_{n},\;t_{n}^{a}\right)$ means agent *n* can begin behavior ${\left(C_{k}\right)}_{k_{1},k_{2}}^{u_{k}}$ after time $t_{n}^{a}$ by providing one of action $a_{\ell }\in A_{n}$. Using these time bounds, we can determine the agents and their providing actions and generate a new party assignment $J^{i},J_{o}^{i}$ to minimize the ending time of subtask *ω_i_*. The efficiency *η* of each assignment $J^{i},J_{o}^{i},\omega_{i}\in \Omega_{u}$ is calculated, and the subtask with maximum efficiency will be chosen. Afterward, the chosen subtask is removed from Ω*_un_* and added to Ω*_as_*. The action sequences $J,J_{o}$ are updated accordingly as $J=J^{i},J_{o}=J_{o}^{i}$ for the next iteration.

### Correctness and completeness analysis

**Theorem 1 (Correctness)** Given two R-posets *P*_1_ = (*Ω*_1_, ⪯_1_, ≠_1_), *P*_2_ = (*Ω*_2_, ⪯_2_, ≠_2_) generated from $B_{1},B_{2}$, we have $L\left(P_{j}\right)\subseteq L\left(P_{1}\right)\cap L\left(P_{2}\right)$, where $P_{j}\in P_{\text{final}}$, $P_{\text{final}}=P_{1}⊗P_{2}$.

**Proof 1** If a word *w* = *σ*′_1_*σ*′_2_⋯ satisfies $P_{j}=\left(\Omega_{j},{⪯}_{j},\neq_{j}\right),P_{j}\in P_{\text{final}}$ it satisfies the three conditions mentioned in Definition 3. In first condition, due to the step Task composition of Poset-prod, we can infer that for any $\omega_{n}^{1}=\left(n,\sigma_{n}^{1},\sigma_{n}^{s1}\right)\in \Omega_{1},$ there exists $\omega_{n}=\left(n,\sigma_{n},\sigma_{n}^{s}\right)\in \Omega_{j}$ with $\sigma_{n}^{1}\subseteq \sigma_{n},\sigma_{n}^{s1}\subseteq \sigma_{n}^{s}$. Thus, we have $\sigma_{i_{1}}^{1}\subseteq \sigma_{i_{1}}\subseteq \sigma \prime_{\ell_{1}}$ and $\sigma_{i_{1}}^{s1}\cap \sigma \prime_{m_{1}}=\emptyset ,\forall m_{1}<\ell_{1}$, which indicates that *w* satisfies *P*_1_ for condition 1. For the second condition, due to Eq. 9 in step Relation update, we have ⪯*_i_* ⊆ ⪯*_j_.* Thus, we can infer that *w* satisfies *P*_1_ for the second condition: Any $\sigma_{i_{1}}^{s1}\cap \sigma \prime_{m_{1}}=\emptyset ,\forall m_{1}<\ell_{1}$, we have (*ω*_*i*_1__, *ω*_*i*_2__) ∈ ⪯*_j_*, thus $\exists \ell_{1}\leq \ell_{2},\sigma_{i_{2}}^{1}\subseteq \sigma_{i_{2}}\subseteq \sigma \prime_{\ell_{2}}$, and $\forall m_{2}<\ell_{2},\sigma_{\ell_{2}}^{s1}\subseteq \sigma_{\ell_{2}}^{s}\subseteq \sigma \prime_{m_{2}}$*.* Additionally, for the last condition, as the word *w* satisfied the ≠*_j_* order of *P_j_.* We have ≠_1_ ⊆ ≠*_j_* due to Eq. 13. Thus, the word w also satisfied the third condition. In the end, we can conclude that *w* satisfies *P*_1_*.* In the same way, we can proof the *w* also satisfies *P*_2_*.* Thus, $L\left(P_{j}\right)\subseteq L\left(P_{1}\right)\cap L\left(P_{2}\right).$

**Theorem 2 (Completeness)** Given two R-posets *P*_1_, *P*_2_ getting from $B_{1},B_{2}$, with enough time budget, Poset-prod returns a set $P_{\text{final}}$ consisting of all final product, and its language $L\left(P_{\text{final}}\right)={⋃}_{P_{i}\in P_{\text{final}}}‍L\left(P_{i}\right)$ is equal to $L\left(P_{\text{final}}\right)=$$L\left(P_{1}\right)⋂‍L\left(P_{2}\right)$*.*

**Proof 2** Due to Theorem 1, it holds that $L\left(P_{\text{final}}\right)\subseteq L\left(P_{1}\right)⋂‍L\left(P_{2}\right)$*.* Thus, we only need to show that $L\left(P_{1}\right)⋂‍L\left(P_{2}\right)\subseteq L\left(P_{\text{final}}\right)$*.* Given a word *w* = *σ*′_1_*σ*′_2_⋯ and $w\in L\left(P_{1}\right)⋂‍L\left(P_{2}\right)$, *w* satisfies the first condition in Definition 3 for both *P*_1_ and *P*_2_ that: $\forall \omega_{i_{1}}^{1}=\left(i_{1},\sigma_{i_{1}}^{1},\sigma_{i_{1}}^{s1}\right)\in \Omega_{1}$*,* there exists *σ*′_*j*_1__ with $\sigma_{i_{1}}^{1}\subseteq \sigma \prime_{j_{1}}$ and $\sigma_{j_{1}}^{s1}\subseteq \sigma \prime_{m_{1}},\forall m_{1}<j_{1}$; $\forall \omega_{i_{2}}^{2}=\left(i_{2},\sigma_{i_{2}}^{2},\sigma_{i_{2}}^{s2}\right)\in \Omega_{2}$, there exists *σ*′_*j*_2__ with $\sigma_{i_{2}}^{2}\subseteq \sigma \prime_{j_{2}}$ and $\sigma_{j_{2}}^{s2}\subseteq \sigma \prime_{m_{2}},\forall m_{2}<j_{2}$*.* If *j*_1_ = *j*_2_, the step in Eq. 8 of Task composition will generate a subtask *ω*_*i*_1__ ∈ *Ω_j_* with $\omega_{i_{1}}⊨\omega_{i_{1}}^{1},\omega_{i_{2}}^{2}$, and $\sigma_{i_{1}}\subseteq \sigma \prime_{j_{1}},\forall m_{3}<j_{1},\sigma_{j_{1}}^{s}\subseteq \sigma \prime_{m_{3}}$*.* If *j*_1_ ≠ *j*_2_, the (7) will generate $\omega_{M_{\Omega }\left(i_{2}\right)}⊨\omega_{i_{2}}^{2}$, with $\sigma_{M_{\Omega }\left(i_{2}\right)}\subseteq \sigma \prime_{j_{2}},\forall m_{4}<j_{2},\sigma_{M_{\Omega }\left(i_{2}\right)}^{s}\subseteq \sigma \prime_{m_{4}}$*.* Thus, there exists $P_{j}\in P_{\text{final}}$ that satisfies the first condition. For the second condition, ⪯*_j_* consisting of two parts generated by Eqs. 9 and 12 guarantees that $w\in L\left(P_{1}\right)\cap L\left(P_{2}\right)$*.* Moreover, Eqs. 10 and 11 guarantee that *w* does not conflict the self-loop constraints of *P*_1_, *P*_2_*.* Thus, the second condition is satisfied. Regarding the third condition, since ≠*_j_* = ≠_1_ ∩ *M_Ω_*(≠_2_) holds in Eq. 13, ≠*_j_* is naturally satisfied by *w*. In conclusion, for any $w\in L\left(P_{1}\right)\cap L\left(P_{2}\right)$*,* $\begin{array}{c} w\in L\left(P_{\text{final}}\right) \end{array}$ holds and vice versa. Thus, $\begin{array}{c} L\left(P_{\text{final}}\right) \end{array}$ is equal to $L\left(P_{1}\right)⋂‍L\left(P_{2}\right)$*.*

## Data Availability

The authors confirm that the data supporting the findings of this study are available within the article.
